# Association between Seminal Vesicle Invasion and Prostate Cancer Detection Location after Transrectal Systemic Biopsy among Men Who Underwent Radical Prostatectomy

**DOI:** 10.1371/journal.pone.0148690

**Published:** 2016-02-05

**Authors:** Young Ik Lee, Hak Min Lee, Jung Ki Jo, Sangchul Lee, Sung Kyu Hong, Seok-Soo Byun, Sang Eun Lee, Jong Jin Oh

**Affiliations:** Department of Urology, Seoul National University Bundang Hospital, Seongnam, Korea; National Health Research Institutes, TAIWAN

## Abstract

**Background:**

Our hypothesis is that the location of the seminal vesicles near the base of the prostate, the more positive cores are detected in the base, the greater the risk of seminal vesicle invasion. Therefore we investigate the clinical outcomes of base dominant prostate cancer (BDPC) in transrectal ultrasound (TRUS) -guided biopsies compared with anteromiddle dominant prostate cancer (AMPC).

**Methods:**

From November 2003 to June 2014, a total of 990 intermediate and high risk prostate cancer (PCa) patients who underwent radical prostatectomy (RP) were enrolled and stratified into two groups according to proportion of positive cores–BDPC group had ≥ 33.3% ratio of positive cores from the prostate base among all positive cores and AMPC group < 33.3% in systemic biopsy. Between two groups, we compared the rate of pathologic outcomes and biochemical recurrence (BCR). We performed multivariate logistic regression model to confirm the significance of BDPC to seminal vesicle invasion (SVI) and Cox proportional hazard analysis to BCR.

**Results:**

Among these 990 PCa patients, the 487 patients in BDPC group had more advanced clinical stage (p<0.001), a higher biopsy GS (p = 0.002), and a higher rate of extracapsular extension (ECE), SVI and BCR (all p<0.001) than AMPC group. The patients in BDPC group had poor BCR free survival rate via Kaplan-meier analysis (p<0.001). The ratio of the base positive cores was a significant predictor to SVI in multivariate analysis (p < 0.001) and significant predictor of BCR in multivariate Cox proportional analysis (hazard ratio: 1.466, p = 0.004).

**Conclusions:**

BDPC in TRUS-guided prostate biopsies was significantly associated with SVI and BCR after adjusting for other clinical factors. Therefore, BDPC should be considered to be a more aggressive tumor despite an otherwise similar cancer profile.

## Introduction

Prostate cancer (PCa) continues to pose significant health care challenges worldwide. Estimates indicate that PCa remains the number one cancer diagnosis in North American and European men, with age-adjusted incidence rates of 85.6 and 59.3 per 100 000, respectively [1]. Many researchers have devoted themselves to the prevention, diagnosis and treatment of PCa. Currently, radical prostatectomy (RP) is one of the gold standards for the treatment of PCa [2]. Several approaches have been used to predict the outcomes of RP among PCa patients, e.g., Partin table [3] and Kattan nomogram [4]. However, few studies have shown the association between the location of tumors detected by transrectal ultrasound (TRUS)-guided prostate biopsies and the patient outcome following RP. TRUS-guided prostate biopsy is one of the most important procedures for the diagnosis of PCa [5–7]. This procedure provides much information, e.g., the number of positive cores that indicate the tumor burden, the ratio of tumor by measuring the tumor length and the core length, the Gleason scores (GS) that is a strong predictive factor of the prognosis and the location of the tumor in the prostate [8].

Seminal vesicle invasion (SVI) of PCa was reported in 7%~24% in some RP series [9–11]. Many studies have reported uniform results of poor prognosis after RP of patients with SVI [12–19]. Our hypothesis is that because of the location of the seminal vesicles near the base of the prostate, the more positive cores are detected in the base, the greater the risk of seminal vesicle invasion because of PCa was multifocal cancer and direct invasion to seminal vesicle might be easier in base PCa in TRUS biopsy. In systematic TRUS-guided prostate biopsies (≥ 12 cores), biopsy cores were obtained from the base, mid and apex portion as same numbers. Therefore the cores from the base portion of prostate which was closed to seminal vesicle due to the direction of ultrasound probe. Therefore, we simply hypothesized that if TRUS-guided prostate biopsies detect positive cores dominantly in the base of the prostate, the likelihood of SVI increases and the overall prognosis worsens. Therefore we studied the associations between the location of prostate cancer in TRUS guided systemic biopsy and the rate of SVI and biochemical recurrence (BCR) after RP.

## Materials and Methods

### Ethics statement

The study was approved by our institutional review board, Seoul National University Bundang Hospital Institutional review board and follows the rules stated in the Declaration of Helsinki. All participants gave written informed consent and were reimbursed for their participation.

### Study population

After obtaining approval by the institutional review board, we retrospectively reviewed a total of 1,772 patients who underwent radical prostatectomy for prostate cancer between November 2003 and June 2014. Among them, we selected the 1,010 patients whose preoperative prostate specific antigen (PSA) was greater than 10 ng/ml or biopsy Gleason score ≥ 7 or clinical stage was higher than T2a (D’amico classification–intermediate and high risk PCa). We excluded patients who underwent adjuvant therapy, other prostate surgery or TRUS-guided prostate biopsy at other institutions. After excluding 20 patients due to incomplete data, the data from a final cohort of 990 patients were collected and analyzed.

### TRUS biopsy protocol and classification

In all patients, prostate biopsy cores were obtained from the base, the mid portion and the apex of the prostate, with at least six biopsy cores per lobe using an automatic firing device. Base dominant prostate cancer (BDPC) was defined when the ratio of positive cores from the base was over one-third of all positive cores according to median value of percentage of base positive core (33.3%). The others which ratio of positive cores from base was less than 33.3% were defined to anteromiddle dominant prostate cancer (AMPC). TRUS-guided prostate biopsy was performed by a specialized radiologist. In each patient, the total number of biopsy cores, the number of positive cores, the maximum tumor length, the percent of tumor length and the GS in biopsy cores were assessed. All TRUS biopsy specimens were processed and evaluated by one expert genitourinary pathologist.

### Outcomes measurement and statistical analysis

The 990 patients were stratified into two groups: BDPC group and AMPC group. The age, body mass index (BMI), serum prostate specific antigen (PSA), prostate volume, clinical stage, biopsy GS sum, total tumor length, total core length, pathology of the RP specimen, extracapsular extension (ECE), SVI, lymph node invasion (LNI), positive surgical margin (PSM), and BCR were analyzed using independent samples t-test and Pearson’s Chi Square test. Magnetic resonance imaging (MRI) study was routinely performed in all PCa patients after prostate biopsy, and these results were taken in consideration in deciding the clinical stage. We also examined which parameters affected SVI following RP using multivariate logistic regression analysis and which parameters affected the BCR using multivariate Cox proportional hazard model. BCR was defined as two consecutive rises in PSA levels of 0.2 ng/ml or higher within at least 2 months following RP. We also examined BCR-free survival using Kaplan-Meier curve analysis. All statistical tests were performed using the Statistical Package for Social Science v.20.0 (SPSS, Chicago, IL, USA).

## Results

### Preoperative and pathologic outcomes after RP between two groups

Among the 990 study patients, 487 patients (49.2%) had BDPC and the other 503 (50.8%) patients had AMPC. Table 1 summarizes the clinical and pathological features of the patients according to aforementioned groups. With regard to the preoperative parameters, when comparing BDPC and AMPC patients, the age, serum PSA level, prostate volume, total core length in biopsy showed no significant differences. However, the BDPC group showed higher BMI (24.61 vs. 24.07 kg/m^2^, respectively, p = 0.002), longer tumor length (2.90 vs 1.81 mm, respectively, p<0.001), higher ratio of tumor extent in biopsy cores (14.87% and 9.35%, respectively, p<0.001) and higher clinical stage (p<0.001) than AMPC group. The BDPC group also showed higher biopsy GS than AMPC group (p = 0.002).

**Table 1:** Patients characteristics and subgroup analysis according to biopsy Gleason score.

Among total patients (n = 990)			
	Base dominant PCa (n = 487)	Anteromiddle dominant PCa (n = 503)	p value
Age, yr, mean(SD)	66.49±6.49	66.92±6.48	0.300
BMI, kg/m2, mean(SD)	24.61±2.66	24.07±2.91	0.002
Preoperative PSA level, ng/ml, mean(SD)	16.59±17.28	16.96±19.90	0.751
Prostate volume, cc, mean(SD)	37.76±17.77	38.15±14.92	0.712
Clinical stage (including MRI), No. of patients(%)			<0.001
T1	226(46.4%)	289(57.5%)	
T2	198(40.7%)	181(36.0%)	
T3	63(12.9%)	33(6.6%)	
Biopsy GS, No. of patients(%)			0.002
≤ 6	63(12.9%)	97(19.3%)	
7	285(58.5%)	302(60.0%)	
8–10	139(28.5%)	104(20.7%)	
Total tumor length in biopsy, mm, mean(SD)	2.90±3.21	1.81±1.83	<0.001
Total core length in biopsy, mm, mean(SD)	19.55±2.01	19.70±1.98	0.253
Ratio of tumor extent in biopsy core, mean(%)(SD)	14.87±16.06	9.35±9.72	<0.001
Pathologic GS, No. of patients(%)			0.001
6	15(3.1%)	31(6.2%)	
7	366(75.2%)	403(80.1%)	
8 ~ 10	106(21.8%)	69(13.7%)	
Extraprostatic extension of tumor, No. patients (%)	239(49.1%)	155(30.8%)	<0.001
Seminal vesicle invasion, No. patients (%)	95(19.5%)	31(6.2%)	<0.001
Positive surgical margin, No. patients (%)	183(37.6%)	165(32.8%)	0.116
Lymph node involvement, No. patients (%)	20(4.1%)	10(2.0%)	0.052
Biochemical recurrence, No. patients (%)	151(31.0%)	97(19.3%)	<0.001
Median postoperative followup duration, months (range)	39(0~126)	36(0~119)	0.131
**The patients with biopsy Gleason score = 7 (N = 587)**	285	302	
Extraprostatic extension of tumor, No. patients (%)	127(60.5%)	83(39.5%)	<0.001
Seminal vesicle invasion, No. patients (%)	34(11.9%)	11(3.6%)	<0.001
Positive surgical margin, No. patients (%)	94(33.0%)	90(29.8%)	0.406
Lymph node involvement, No. patients (%)	8(2.8%)	5(1.7%)	0.343
Biochemical recurrence, No. patients (%)	66(23.2%)	58(19.2%)	0.241
**The patients with biopsy Gleason score = 8~10 (N = 243)**	139	104	
Extraprostatic extension of tumor, No. patients (%)	97(69.8%)	58(55.8%)	0.025
Seminal vesicle invasion, No. patients (%)	59(42.4%)	19(18.3%)	<0.001
Positive surgical margin, No. patients (%)	75(54.0%)	54(51.9%)	0.753
Lymph node involvement, No. patients (%)	12(8.6%)	5(4.8%)	0.247
Biochemical recurrence, No. patients (%)	75(54.0%)	36(34.6%)	0.003

PCa = prostate cancer; BMI = Body mass index; PSA = prostate-specific antigen; GS = Gleason score

After RP, BDPC group showed higher rate of ECE (49.1% vs. 30.8%, p < 0.001), SVI (19.5% vs. 6.2%, p < 0.001) than AMPC group, respectively. There were no significant differences of PSM and LNI between two groups. Stratified to biopsy GS, ECE and SVI were also higher in BDPC group than AMPC group. Among the patients whose biopsy GS 7, 34 BDPC patients (11.9%) and 11 AMPC patients (3.6%) had SVI (p<0.001). Among the patients whose biopsy GS 8–10, 59 BDPC patients (42.4%) and 19 AMPC patients (18.3%) had SVI (p<0.001).

### The effect of ratio of positive cores in base to seminal vesicle invasion

In a multivariate logistic regression analysis to identify the factors associated with SVI, preoperative serum PSA level (odds ratio (OR) = 1.011, 95% confidence intervals (CI) 1.005–1.016, p < 0.001), biopsy Gleason score (OR = 2.411, 95% CI 1.843–3.102, p<0.001), ratio of tumor extent in biopsy (OR = 2.512, 95% CI 1.521–4.025, p<0.001), total number of positive core (OR = 1.597, 95% CI 1.275–2.000, p < 0.001) and ratio of base positive cores (OR = 1.512, 95% CI 1.030–2.183, p = 0.001) were found to be independent predictive factors of SVI following RP (Table 2). To reduce the bias of biopsy GS, we performed subgroup multivariate analysis according to biopsy GS. The ratio of base cores also significant predictor to SVI (OR = 3.060, 95%CI 1.579–5.932, p = 0.001) among only the patients with biopsy GS 7 (n = 587) and (OR = 3.439, 95%CI 1.686–7.013, p = 0.001) among the patients with biopsy GS 8–10 (n = 243).

**Table 2:** Multivariate logistic regression analysis to predict seminal vesicle invasion after radical prostatectomy among total cohort.

	Odds ratio	95% CI	p value
**Among total patients**			
Age	1.412	0.951–1.081	0.937
Serum PSA level	1.011	1.005–1.016	<0.001
Clinical stage	1.211	0.977–1.517	0.063
Biopsy Gleason score	2.411	1.843–3.102	<0.001
Prostate size	1.004	0.995–1.017	0.202
Ratio of tumor extent in biopsy cores (%)	2.512	1.521–4.025	<0.001
Total numbers of positive cores (<6 vs 6≤ cores)	1.597	1.275–2.000	<0.001
Ratio of prostate base positive cores (%)	1.512	1.030–2.183	0.001
**The patients with biopsy Gleason score = 7**			
Age	1.012	0.964–1.064	0.624
Serum PSA level	1.028	1.008–1.049	0.006
Clinical stage	1.230	0.627–2.412	0.547
Prostate size	1.008	0.993–1.023	0.313
Ratio of tumor extent in biopsy cores (%)	2.175	1.342–3.085	<0.001
Total numbers of positive cores (<6 vs 6≤ cores)	1.124	1.004–1.259	<0.001
Ratio of prostate base positive cores (%)*	3.060	1.579–5.932	0.001
**The patients with biopsy Gleason score = 8–10**			
Age	1.043	0.981–1.109	0.175
Serum PSA level	1.020	1.006–1.035	0.006
Clinical stage	1.318	0.623–2.788	0.471
Prostate size	1.021	0.996–1.046	0.098
Biopsy Gleason score	2.441	1.743–3.132	<0.001
Ratio of tumor extent in biopsy cores (%)	2.460	1.821–3.325	<0.001
Total numbers of positive cores (<6 vs 6≤ cores)	1.340	1.195–1.502	<0.001
Ratio of prostate base positive cores (%)*	3.439	1.686–7.013	0.001

*Multivariate logistic regression after adjusting aforementioned parameters

### The effect of ratio of positive cores in base to biochemical recurrence

**Fig 1:**
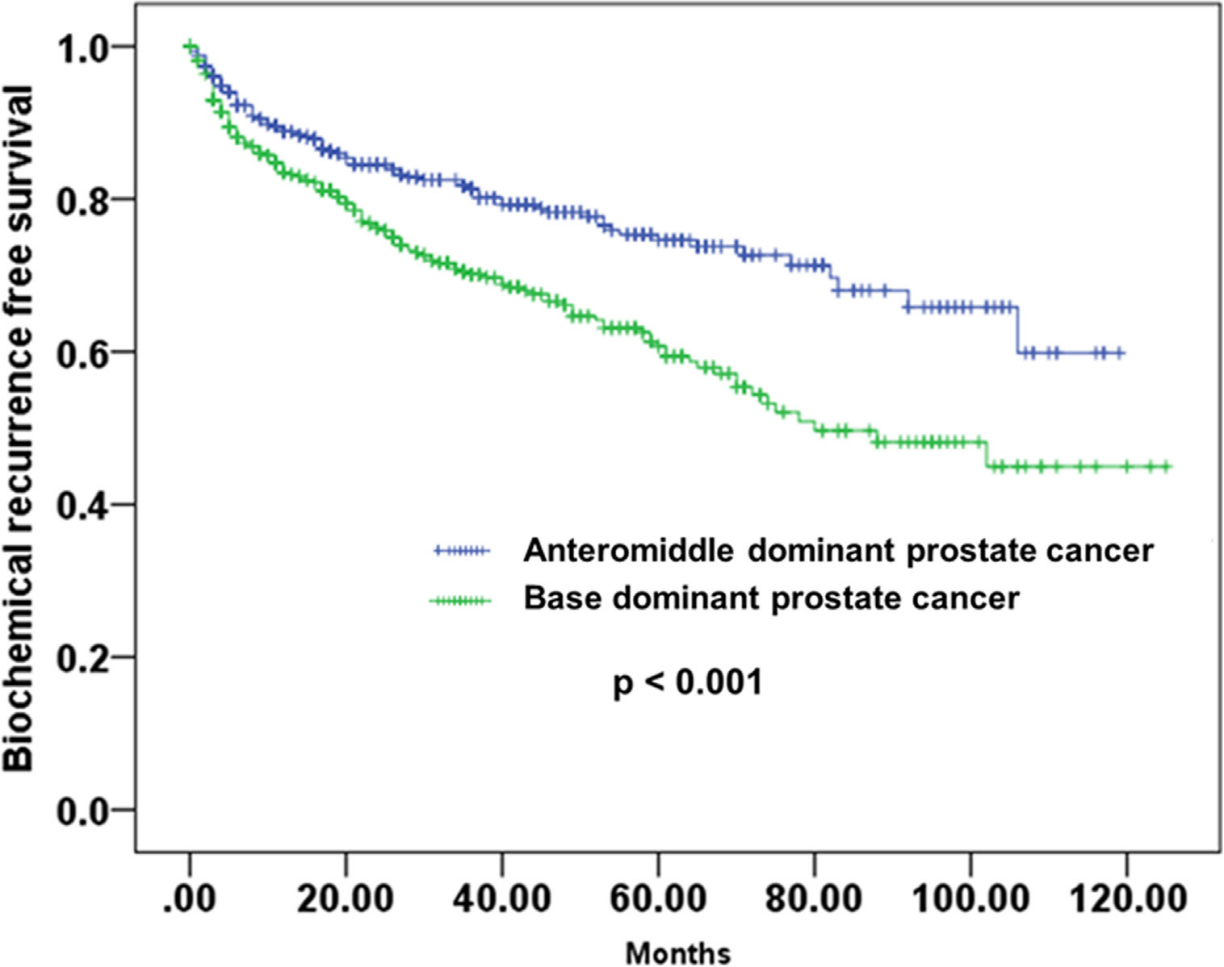
Kaplan-Meier survival curve of biochemical recurrence-free survival according to ratio of base cores. The 5-year BCR-free survival was 60.7% in the base dominant prostate cancer group compared with 74.6% in the anteromiddle dominant prostate cancer group (log rank test, p-value = 0.001).

During the median follow-up period of 37 months, BCR was occurred in 151 patients (31.0%) among BDPC group and 97 patients (19.3%) among AMPC group. We compared the BCR-free survival using the Kaplan-Meier survival curve and found a significant difference of BCR- free survival between these two groups (log rank test, p<0.001) (Fig 1). The 5-year BCR-free survival was 74.6% in the BDPC group and 60.7% in AMPC group.

**Fig 2:**
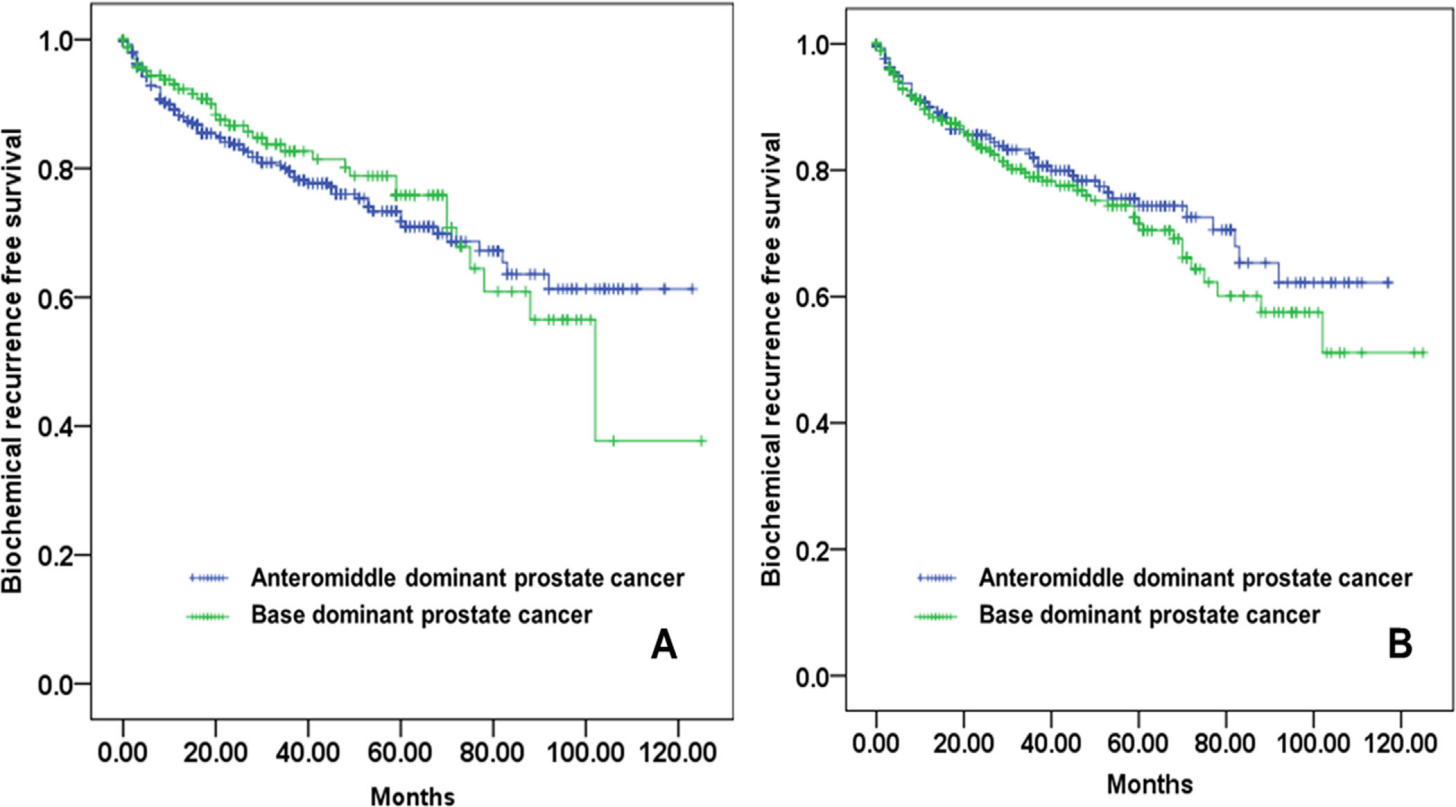
Kaplan-Meier survival curve of biochemical recurrence-free survival according to ratio of base cores (A) among patient with biopsy Gleason score 7 (log rank test, p-value = 0.074) and (B) among patients with biopsy Gleason score 8–10 (log rank test, p-value = 0.004).

As described in Table 3, multivariate Cox proportional hazard model to predict BCR showed that preoperative serum PSA levels (HR = 1.011, 95% CI 1.00–1.016, p<0.001), biopsy Gleason scores (HR = 2.411, 95% CI 1.843–3.102, p<0.001), ratio of tumor extent in biopsy scores (HR = 2.512, 95% CI 1.521–4.025, p<0.001), total number of positive core (HR = 1.597, 95% CI 1.275–2.000, p < 0.001) and ratio of base positive cores (HR = 1.512, 95% CI 1.030–2.183, p = 0.001) were independent predictive factors of BCR after RP. When the patients were divided according to biopsy GS, the ratio of base positive cores was not independent predictive factors of BCR among patients with biopsy GS 7, but independent factors in biopsy GS 8–10 (HR = 1.810, 95% CI 1.315–2.482, p = 0.004). As shown in Fig 2, BCR-free survival analysis using the Kaplan-Meier survival curve in patients with biopsy GS 7 (Fig 2A) were not significant according to ratio of positive cores, but in patients with biopsy GS 8–10 (Fig 2B) were significant (p = 0.004).

**Table 3:** Multivariate Cox proportional hazard model to predict biochemical recurrence after radical prostatectomy using preoperative factors.

	Hazard ratio	95% CI	p value
**Among total patients**			
Age	1.412	0.951–1.081	0.937
Serum PSA level	1.011	1.005–1.016	<0.001
Clinical stage	1.211	0.977–1.517	0.063
Biopsy Gleason score	2.411	1.843–3.102	<0.001
Prostate size	1.004	0.995–1.017	0.202
Ratio of tumor extent in biopsy cores (%)	2.512	1.521–4.025	<0.001
Total numbers of cores (<6 vs 6≤ cores)	1.597	1.275–2.000	<0.001
Ratio of prostate base positive cores (%)	1.512	1.030–2.183	0.001
**The patients with biopsy Gleason score = 7**			
Age	0.997	0.971–1.024	0.827
Serum PSA level	1.021	1.014–1.029	<0.001
Clinical stage	1.484	1.019–2.161	0.040
Prostate size	1.003	0.993–1.012	0.569
Ratio of tumor extent in biopsy cores (%)	1.067	0.714–1.592	0.752
Total numbers of cores (<6 vs 6≤ cores)	1.066	1.000–1.136	0.049
Ratio of prostate base positive cores (%)*	1.085	0.525–1.617	0.074
**The patients with biopsy Gleason score = 8–10**			
Age	1.006	0.969–1.044	0.767
Serum PSA level	1.005	0.999–1.011	0.114
Clinical stage	1.187	0.771–1.830	0.436
Biopsy Gleason score	2.311	1.243–2.422	<0.001
Prostate size	1.004	0.991–1.018	0.566
Ratio of tumor extent in biopsy cores (%)	1.469	0.980–2.204	0.063
Total numbers of cores (<6 vs 6≤ cores)	1.134	1.066–1.205	<0.001
Ratio of prostate base positive cores (%)*	1.810	1.315–2.482	0.004

* Multivariate Cox proportional hazard analysis after adjusting aforementioned parameters

## Discussions

We demonstrated that BDPC was significantly associated with SVI, ECE and BCR after RP. After adjusting other confounding factors, the ratio of positive core from base was important factors to predict SVI and BCR in multivariate logistic and Cox proportional analysis. Main finding in this study, BDPC had higher rate of SVI.

The SVI in PCa is an important prognostic factor for recurrence after RP [12–19]. D’Amico et al. [12] reported that the 2-year actuarial PSA failure rates were 84% versus 23% in patients with and without SVI, respectively. Debras *et al*. [14] analyzed 52 patients whose clinical stage T3b N0 M0 indicated that SVI was present and found that the overall 5-year PSA progression free rate was only 14.4%. All these studies suggest that predicting the presence of SVI before RP is important for surgical planning and management after RP.

Disease models have been developed to predict the outcomes after RP. Partin nomograms [3] that combine preoperative serum PSA level, clinical (TNM) stage, and biopsy Gleason score have been widely used to determine the likelihood of various final pathologic stages at RP and predict the probabilities of organ confined disease, ECE, SVI and LNI. Kattan nomograms [4] that incorporate pretreatment variables (clinical stage, biopsy GS, serum PSA and the amount of cancer in a systematic biopsy specimen) can predict the probability that a man with PCa has an indolent tumor and can be considered for active surveillance. These nomograms to predict the final pathology have been updated but none of these has taken into account the tumor locations in TRUS-guided prostate biopsies as a predictive value. In our institution, at least 12 core systematic TRUS-guided prostate biopsies were performed using a mapping system whereby 12 cores included almost all prostate area covering the apex, the mid portion and the base area both medially and laterally. With information about the locations of the tumors, we hypothesized that the location of tumor could be an important factor in PCa outcomes, and we found the BDPC had higher rate of SVI. Due to high level of SVI, BDPC also had higher rate of BCR compared with AMPC.

Cancer itself has an ability to invade adjacent tissue and organ. Some reports suggested that cancer spread by direct extension in which a tumor growing in a body cavity releases cells or fragments that can seed serosal and/or mucosal surfaces and develop into new growths [20,21]. Another gastrointestinal cancer also tends to invade into adjacent organs and the anatomic site of gastric cancer is known to be one of the clinically important factors in the evaluation of the pathology of original cancer as it crosses the serosal layer and extends to adjacent organs [22]. Therefore we simply expected that the high cancer volume closed to seminal vesicle had higher chance to invasion to seminal vesicle in PCa, we found this association in our retrospective study.

Ohori M *et al*. [23] analyzed 312 RP specimens obtained from patients with T1-T3 PCa. They found three types of SVI. Type I involvement was through direct spread along the ejaculatory duct complex into the seminal vesicles. Type II involvement consisted of tumor spread outside of the prostate, through the capsule, and into the seminal vesicle. Type III involvement was characterized by the finding of isolated deposits of cancer cells in the seminal vesicles with no contiguous primary cancer in the prostate. Type II involvement was found in 61% of patients (Type II only was in 33% of patients and combination of Type I and Type II was 28%). The higher rate of type II indicates the higher likelihood in SVI that cancer cells penetrate the capsule and invade the seminal vesicle directly. Close anatomical location is essential such as the prostate base area and seminal vesicles is essential in this process.

Villers *et al*. [9] reported that the route of seminal vesicle invasion from the prostate in 46 cases involved direct tumor spread into the midbase region near the ejaculatory ducts. Guillonneau *et al*. [11] analyzed 75 patients with localized PCa and reported that the risk of SVI was 0 (0/21 patients) when the 2 base prostatic biopsies were negative, 10.25% (4/39 patients) when 1 of the 2 base prostatic biopsies was positive and 73.33% (11/15 patients) when both base prostatic biopsies were positive. This report is consistent with our data that BDPC is associated with a higher rate of SVI. Koh *et al*. [24] investigated the nomogram to predict SVI based on the extent and location of cancer in systematic biopsy results. They evaluated patients with T1c-T3 PCa who had RP and concluded that the presence and amount of cancer in systematic needle biopsy cores from the base of the prostate strongly predicts the presence of SVI. They reported 12.8% of SVI when the cancer was present in a biopsy core from the base compared to only 1.2% when the cancer was not present in base core. They also reported that serum PSA, GS and percent cancer at the base were the only significant predictors of SVI. Similarly in our study, serum PSA levels, biopsy GS, ratio of tumor extent in biopsy cores and ratio of base positive cores were independent predictors of SVI. Their results were similar to ours in that BDPC resulted in a higher rate of SVI. However, these authors obtained more than 6 TRUS-guided prostate biopsy cores per patients compared to more than 12 cores in our institution. Moreover, we found that BDPC correlated with BCR.

Many studies have investigated the accuracy of MRI in local staging such as SVI, their results were heterogenous and it driving the ongoing debate regarding the usefulness of MRI and the best imaging protocol for PCa staging. Recent meta-analysis showed that MRI had high specificity for local PCa staging and improved sensitivity for SVI detection [25]. In this study, MRI was performed in all patients, however MRI was conducted after histological confirmation of prostate cancer during prostate biopsy due to countries insurance policy. Therefore we did not use MRI finding in our analysis because of MRI was conducted after biopsy, it had some radiologic bias from hemorrhage or inflammation. And we simply wanted to know the effect of tumor location to adjacent related organ without such expensive examination.

Our current study presents some limitations. First, data in this study were obtained retrospectively. Second, a comparison of the dominant tumor location in TRUS biopsy specimens with the actual dominant location of the tumor in RP specimens would have been preferable because of the possibility of sampling error information about the latter is not available. Third, there was not accurate landmark to classify the location of prostate–apex, mid and base during TRUS guided prostate biopsy. Although all prostate biopsies were performed much experienced specialized uro-radiologists, it could be limitation. Another limitation was not equally distributed in biopsy GS between two groups, BDPC group had higher biopsy GS. Although we adjusted this limitation by performing the subgroup analysis according to biopsy GS and adjusted the factor of GS in multivariate analysis, this should be overcome in future prospective study. Another preoperative option to predict locally advancement using MRI/ultrasound funsion biopsy might be answer as a tool for the clinical setting in future study. Although these limitations, we found the direct cancer effect to SVI in PCa, this study might be an important message to real clinical situation about careful management of BDPC.

## Conclusions

The ratio of tumor extent from the base of the prostate detected in TRUS-guided prostate biopsies strongly predicts the presence of SVI. Moreover, this ratio can predict the BCR after RP in PCa. Together with the serum PSA and biopsy GS, the dominant site of PCa should be taken into consideration as a predictive factor of the prognosis of PCa after RP, therefore we should be careful to treat BDPC in TRUS guided biopsy.
